# Inter-relationship between microsatellite instability, thymidylate synthase expression, and p53 status in colorectal cancer: implications for chemoresistance

**DOI:** 10.1186/1471-2407-6-150

**Published:** 2006-06-05

**Authors:** Sanjay Popat, Richard Wort, Richard S Houlston

**Affiliations:** 1 Section of Cancer Genetics, Institute of Cancer Research, Sutton, Surrey, SM2 5NG, UK

## Abstract

**Background:**

Studies indicate that thymidylate synthase (TS) expression, p53 and mismatch repair status have potential to influence colorectal cancer (CRC) outcome. There is, however, little data on the inter-relationship between these three markers. We sought to investigate whether relationships exist between these markers that might contribute to CRC phenotypes.

**Methods:**

Four hundred and forty-one stage I-III CRCs were investigated. p53 status and TS expression were assessed by standard immunohistochemistry methods. Mismatch repair status was determined by assessment of microsatellite instability (MSI) using radiolabelled microsatellite genotyping.

**Results:**

244 tumours (55%) over-expressed p53, and 259 (58%) expressed high TS levels. 65 tumours (15%) had MSI. A significant relationship between p53 over-expression and high TS expression was observed (p = 0.01). This was independent of MSI status. A highly significant inverse relationship between MSI and p53 status was observed (p = 0.001). No relationship was seen between MSI status and TS expression (p = 0.59).

**Conclusion:**

Relationships exist between p53 status and TS expression, and MSI and p53 status. These inter-relationships may contribute to the clinical phenotype of CRCs associated with each of the molecular markers. High TS expression is unlikely to account for the clinical behaviour of CRCs with MSI.

## Background

Colorectal cancer (CRC) is one of the commonest malignancies of developed countries [1], with approximately 19,000 and 160,000 new cases in the UK and US, respectively, each year, and around 500,000 new cases world-wide [2,3]. CRC tumourigenesis is a multi-step phenomenon, typified by a series of genomic events that parallel development of invasive malignancy from normal epithelium through formation of pre-malignant adenomas [4]. Whilst most (~85%) CRCs develop through the chromosomal instability pathway, in which adenoma formation is typified by loss of APC function, and development of invasive malignancy by *TP53 *mutation [4], a smaller number (~15%) develop as a consequence of mismatch repair (MMR) deficiency. These tumours are characterised by high frequency microsatellite instability (MSI), proximal colonic distribution, poor differentiation, mucinous appearance, and lymphocytic infiltration [5]. Additionally, these tumours tend to retain the native diploid state [5]. By contrast, chromosomally unstable tumours tend to be aneuploid and have no site predilection [6].

Several studies have demonstrated that tumour molecular phenotype is a determinant of CRC prognosis [7-9]. Although many markers of prognosis have been investigated in recent years, the most promising to date are mismatch repair status, thymidylate synthase (TS) expression level, and p53 status.

Tumours developing through the mismatch repair pathway are characterised by loss of mismatch repair gene function (primarily *hMLH1*), through either mutation or more commonly, epigenetic change [5]. This results in somatic hypermutability most pronounced at short tandem repeat sequences such as microsatellites, termed microsatellite instability (MSI), readily detectable by the presence/absence of novel alleles or the shortening of at least 2–3 repeat units by electrophoresis. Several studies of clinical datasets have indicated that CRCs with MSI are associated with improved survival [10]. However, *in vitro *data has indicated that these tumours are paradoxically characterised by 5FU chemoresistance [11,12]. Although the mechanism for these observations remain unclear, the notion that fluoropyrimidine sensitivity might be modulated through either TS expression or p53 mutation is plausible [13]. Thymidylate synthase (TS) is a major protein involved in CRC development and outcome. As well as providing the sole intracellular source of thymidine for DNA synthesis [14], TS is also a target for a number of drugs used for CRC treatment, including 5-fluorouracil (5FU), whose mechanism of action is primarily mediated through competitive TS inhibition. Thymidylate synthase expression has been shown to be a key determinant of 5FU resistance *in vitro *[14,15] and several patient series have confirmed poorer outcomes in those with tumours expressing high TS levels [16]. Mutation in the tumour suppressor *TP53 *has also been associated with chemoresistance and poorer survival in CRC [17,18]. *TP53 *maps to chromosome 17p13.1 [19] and is one of the commonest genes mutated in CRC, encoding a transcription factor (p53) critically involved in control of cell cycle, apoptosis, and carcinogenesis [20-25].

Whilst each of these markers impact on CRC prognosis, the inter-relationship between each has been the subject of few studies. Hence, the contribution to the clinical behaviour of CRCs associated with either p53, TS or mismatch repair status of any such inter-relationship is unclear. Here we report analysis of the largest series of early stage CRC assessed for these three clinically relevant molecular co-variates.

## Methods

### Patients

Four hundred and forty-one paired paraffin-embedded formalin fixed tissue blocks (one tumour tissue and one normal tissue) from patients with stage I-III CRC collected at the time of potentially curative surgery were assessed. The study was conducted in accordance with the tenets of the Declaration of Helsinki.

Fifteen-micron sections of formalin-fixed paraffin embedded tumours were cut onto double-sided clear adhesive tape on glass slides. Regions of ≥ 70% tumour were microdissected after light staining with toluidine blue. Ten-micron whole sections of normal mucosa from a separate paired block of normal colorectal mucosa from each patient were provided as a source of germline DNA extraction. DNA was extracted using standard commercially available methods (Qiagen, West Sussex, UK) and genotyping was performed at the highly sensitive and specific mononucleotide microsatellite locus BAT26 [26,27]. In cases where no paired normal tissue was available, tumour DNA was genotyped alone. Target DNA sequences were amplified using ^32^P end-labelled primers. Mismatch repair status was assigned as MSI or microsatellite stable (MSS) by presence or absence of novel alleles or the shortening of at least 2–3 repeat units at autoradiography. Genotyping was performed at least twice per sample. Only tumours with unambiguous genotypes were assigned MSI status.

### p53 and TS evaluation

Three micron sections from a representative part of the primary tumour were cut onto silane coated glass slides and assessed for TS and p53 expression by the avidin-biotin complex immunohistochemical technique (Vectastain Elite ABC kit, Vector Laboratories Inc, Burlingame, CA, USA) [28]. Negative and positive control slides were included in each staining run. Primary antibody was replaced with phosphate buffered saline, pH 7.6/0.1% (v/v) Tween solution for negative controls, and tumour sections known to stain heavily for target antigens were used for positive controls.

Tumour sections were deparaffinized in Histoclear (National Diagnostics, Hull, UK) and hydrated in decreasing concentrations of ethanol. Endogenous peroxidase activity was quenched with 5% hydrogen peroxide in methanol for 20 minutes. Antigen retrieval was required for p53 only and was by a microwave oven-based method. Specifically, sections were incubated in boiling 10 mmol/L citric acid buffer (pH 6.0) for 10 minutes and then cooled in running water. For both TS and p53, non-specific background staining was blocked with 20% goat serum for 20 minutes. Sections were then incubated with appropriate primary antibodies at a 1:100 dilution, in a humidified chamber at room temperature for 60 minutes, using either a validated rabbit polyclonal antibody to recombinant human TS [29], or an anti-p53 mouse monoclonal (clone DO-7, Dako Corp, Denmark). After rinsing, a biotinylated anti-rabbit secondary antibody was applied for 30 minutes followed by further washing, and avidin-biotin-peroxidase complexes (Vectastain Elite ABC kit; Vector Laboratories Inc, Burlingame, CA, USA). Immunostaining was developed by applying freshly prepared 0.05% 3, 3'-diaminobenzidine tetrahydrochloride (Vectastain Elite ABC kit, Vector laboratories). Sections were counterstained in Mayer's Haematoxylin (Sigma Chemical Co., St Louis, MO, USA), dehydrated in a series of ethanols, cleared in Histoclear (National Diagnostics, Hull, UK) and mounted with glass coverslips using DePeX (BDH, Poole, UK).

### Immunohistochemistry evaluation

All slides were randomly allocated for independent assessment by two observers (RSH and SP), blinded to marker status. TS expression was assessed using the commonest reported method; a semiquantative grading of tumour tissue for chromagen intensity from 0 to 3, with the highest tumour staining detected as the reference for classification. Grades 0 and 1, representing none and minimal staining respectively, were defined as the "low" expression group, whereas grades 2 and 3 were defined as the "high" expression group. p53 immunoreactivity was dichotomised into positive or negative based on staining of malignant nuclei, with a threshold of 10%.

Level of scoring agreement between the two observers was recorded. In cases of disagreement, marker status was discussed and determined by consensus.

### Statistical analysis

All statistical manipulations were preformed using STATA (Version 7, Stata Corp. TX77840, USA). Relationships between TS, p53 expression, and MSI status were assessed with Fisher's exact test, stratified by relevant marker.

## Results

### Tumour phenotypes

**Figure 1:**
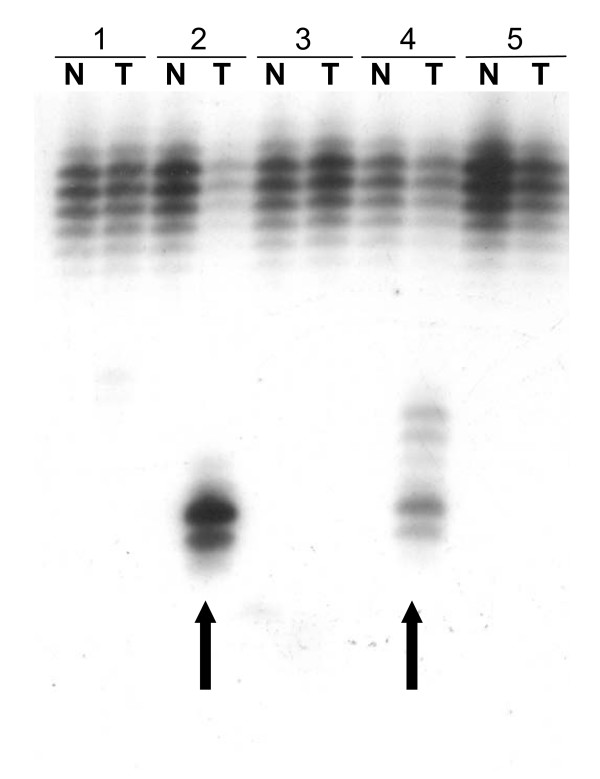
Representative examples of microsatellite instability (MSI). Somatic (T lanes) and paired germline (N lanes) genotypes at the microsatellite marker BAT26 detected by autoradiography in 5 normal/normal pairs are shown. New somatic alleles (lanes T) (tumours 2 and 4) compared to germline genotypes (lanes N), as indicated by the arrows, are indicative of MSI.

Genotype data was available from all 441 specimens. Sixty-five (15%) tumours demonstrated MSI, whereas 376 had MSS. Figure 1 shows representative tumour genotypes, with two examples of MSI.

**Figure 2:**
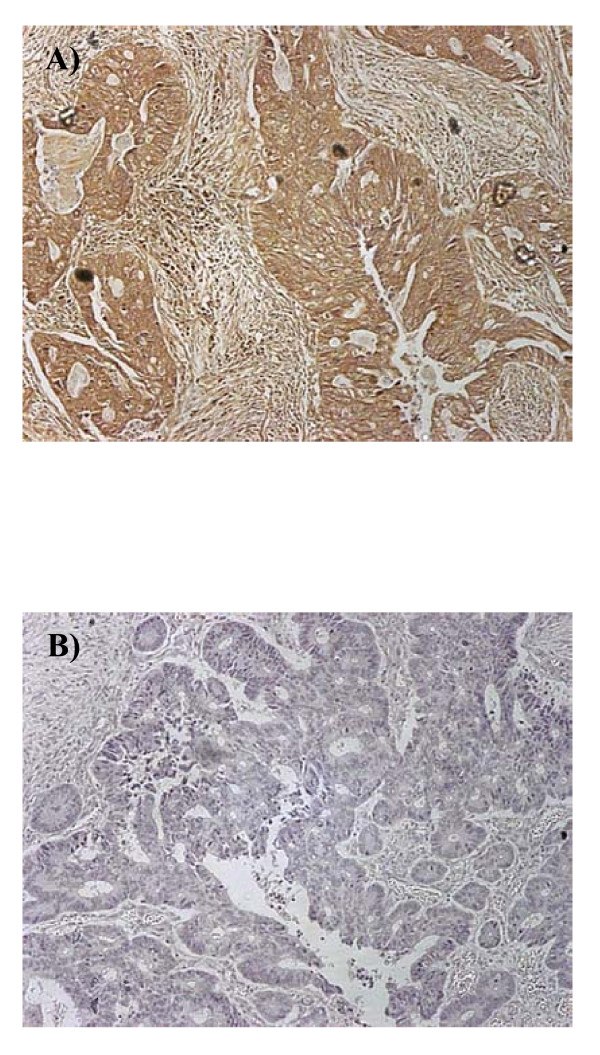
Immunohistochemical analysis of tumours for thymidylate synthase (TS) showing (a) high level, and (b) low level TS expression.

**Figure 3:**
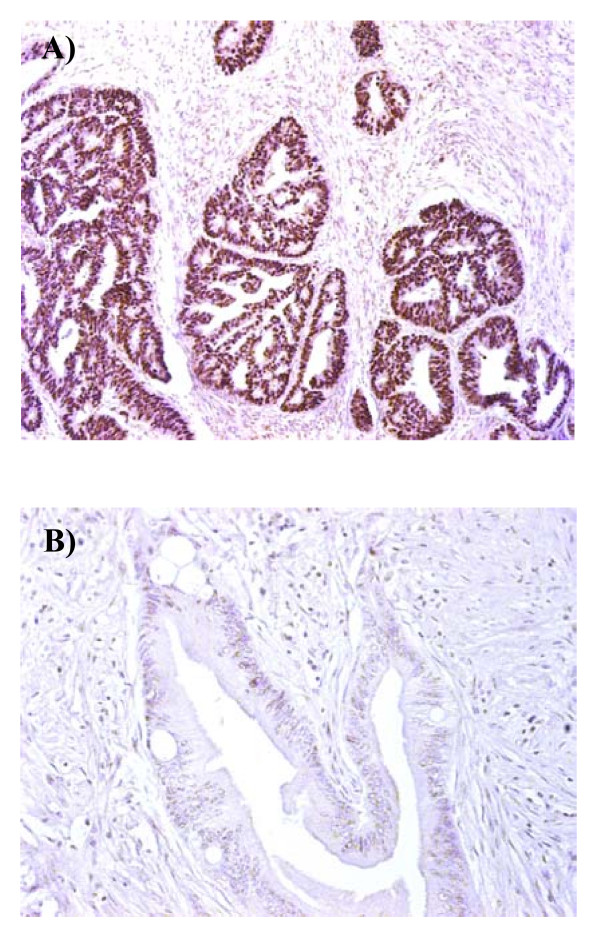
Immunohistochemical analysis of tumours for p53 (a) p53 over-expression, (b) no p53 over-expression.

One quarter of the tumours stained for TS with grade 3 (101, 23%) or grade 0 (15, 3%) levels of chromagen intensity, whilst three-quarters stained with either grade 1 (167, 38%) or grade 2 (158, 36%) intensity. When dichotomised into high and low levels of TS expression, just over half of the samples expressed high TS levels (259, 59%), with the remainder having low-level expression (182, 41%). Just over half demonstrated p53 over-expression (244, 55%), with the remainder showing neither over-expression nor staining (197, 45%). Figures 2 and 3 show representative sections stained for TS and p53, respectively, demonstrating levels of chromagen intensity required to allocate expression status.

### Inter-relationship between p53, TS, and mismatch repair status

A significant association between p53 status and TS chromagen intensity was observed (p = 0.01). This was maintained when dichotomising TS expression into high and low groups. Specifically, tumours with p53 over-expression were significantly associated with high TS levels (p = 0.005, Table 1). When stratified by MSI status, a relationship between p53 status and TS level was again observed (p = 0.005), with tumours over-expressing p53 (p53 positive) tending to express high TS levels. This relationship was maintained in both subsets of tumours with MSS (p = 0.01, Table 1), and MSI (p = 0.30, Table 1). Although this relationship did not reach formal statistical significance in MSI tumours, this was likely due to the small numbers of tumours assessed, since an over-representation of p53 positive tumours with high TS levels was again observed.

As expected, a highly significant inverse relationship between mismatch repair status and p53 status was observed; MSI tumours tending to be p53 negative (p = 0.001, Table 2). This was maintained and not influenced by TS status (Table 2).

No relationship was observed between mismatch repair status and TS level (p = 0.59, Table 3) and p53 status did not impact on these findings (Table 3).

In order to exclude any bias that may have resulted from erroneous classification, we reassessed p53 status using thresholds of 5% and 15%. This resulted in no reclassification, and therefore no change in associations. Our results were further re-analysed using the Pearson χ^2 ^test. All significant and non-significant associations were maintained. Our results have therefore been reported using the Fisher test, which gives the exact p value, rather than the asymptotic value calculated by the χ^2 ^test.

## Discussion

We have investigated the relationship between MSI, TS and p53 status using standard genotyping and immunohistochemical methods in early stage CRC. Although only loosely correlating with *TP53 *mutation, p53 nuclear over-expression detected by IHC has been found to be a marker of worse prognosis in many previously published analyses of CRC datasets [21,30,31]. Our results indicate a highly significant association between p53 status and TS expression, with CRCs expressing high TS levels more likely to over-express p53, regardless of MSI status.

In normal cells, regulation of both TS and p53 are independently tightly controlled. In addition to it's role in enzyme catalysis, TS also functions as a RNA binding protein [32,33], regulating it's own expression by a negative autoregulatory mechanism [33,34], as well as binding to it's own RNA, to form TS-ribonucleoprotein complexes with several RNA species including *c-myc *and *TP53 *[35]. Although *in vitro *data indicates that p53 and TS have the ability to regulate each-other in non-malignant cells [32,33,36], evidence for a relationship in CRC has been conflicting, with some studies reporting that TS negatively regulates p53 expression [37], whilst others have shown no such relationship [38,39]. In addition, the potential for wild-type p53 to regulate TS expression has also been demonstrated using a luciferase-based system, which showed that p53 expression can inhibit *TYMS *promoter activity [40]. These results are, however, based on *in vitro *analysis, and no studies have characterised the role of mutant p53 or whether aberrant mismatch repair impacts on the relationship. Our results suggest a relationship between *TP53 *status and TS expression implying that the poor prognosis and chemoresistance observed in studies of CRC patients with either high TS expression or *TP53 *mutation/p53 over-expression, may have been impacted on by either co-variate.

A number of potential mechanisms may account for our findings. *TP53 *mutation is associated with loss of transcriptional activity control resulting in up- or down-regulation of downstream p53 effectors. Thus, inactive p53 might disinhibit an inhibitory role of p53 on TS expression. Alternatively, according to the gain-of-function paradigm [41], mutant p53 might acquire novel activities that promote cell growth and survival [42], perhaps through enhanced TS expression. An example of the latter case is the 273 Arg-His mutation, which has strong transactivating activity [43]. Although the role of this specific mutation in regulating TS expression is unknown, it is feasible that specific p53 mutations retain transcriptional regulatory activity, which may be partially responsible for control of transcriptional activity of proteins such as TS. Supporting this, Lenz et al. investigated the relationship between *TP53 *mutation and TS expression in a series of 36 CRCs and demonstrated that CRCs with p53 mutations affecting the zinc-binding domains had higher TS expression than those with mutation outside these domains [44]. Zinc domains are involved in direct DNA contact, protein stabilization, and structural activity, indicating that these mutations may have a severe impact on the transcriptional activity of p53.

Nine other studies have investigated the relationship between p53 and TS expression, based on immunohistochemistry, using p53 over-expression as a surrogate of *TP53 *mutation [8,45-52]. However, most have been based on small sample sizes (median 66, range 22[51]–691[48]). Our results are consistent with four [44-46,50] of these studies, which also demonstrated a relationship between p53 and TS expression. Moreover, our study is the only one to assess this relationship stratified by MSI status. The relationship between p53 and TS was observed both in tumours with MSI and those with MSS, implying that aberrant mismatch repair is unlikely to impact on any mechanism relating p53 to TS expression. However, given the small number of samples with MSI, this cannot be entirely excluded.

As expected a highly significant inverse relationship between p53 status and MSI was observed. This relationship was independent of TS status, observed in both tumours expressing low and high TS levels (p ≤ 0.03), and is consistent with the concept that most CRCs develop either along the chromosomal instability pathway associated with *TP53 *mutation and MSS tumours or the aberrant mismatch repair pathway associated with wild-type p53 and MSI tumours [53-56]. This association may also explain why tumours with MSI seem to have an improved prognosis compared to those with intact mismatch repair. However, we demonstrated no relationship between MSI and TS status. This finding was independent of p53 status. Whilst the precise mechanisms by which cells with MSI seem resistant to 5FU *in vitro *has been poorly defined, Ricciardiello et al. [57] have suggested that this may, in part, be due to TS over-expression TS. In their analysis of 192 CRCs the authors demonstrated an association between CRCs with MSI and high TS expression [57]. This observation was, however, contrary to an earlier study based on an analysis of 53 CRCs, which also observed no relationship between TS expression and the MSI phenotype [58]. Moreover, the study by Ricciardiello et al. [57] was based on only 24 CRCs with MSI and the rate of high TS expression in their study was at the lower end of that previously reported (21%) [16] and may have biased findings. Our data, based on a sample size over two times larger, precludes at least a 16% difference between MMR status and high TS expression at the 5% threshold, with 90% power.

Our results provide little evidence that TS expression plays a major role in defining chemoresistance in microsatellite unstable CRCs, but gives support to previous reports of an inverse relationship between MSI and p53 status. In addition, we demonstrated that TS expression is related to p53 status, and this relationship may in part account for the poorer prognosis and relative chemoresistance seen in these tumours.

## Conclusion

Relationships exist between TS expression and p53 status, and MSI and p53 status in CRC that may account for the clinical phenotypes of these tumours. High TS expression is unlikely to play a major role in the clinical behaviour of CRCs characterised by MMR deficiency.

## Competing interests

The author(s) declare that they have no competing interests.

## Authors' contributions

RW and SP carried out the molecular and immunohistochemical studies. SP and RSH reviewed results and assigned tumour categories. RW participated in study design. SP and RSH conceived the study, performed the statistical analysis, and drafted the manuscript. All authors read and approved the final manuscript.

## Pre-publication history

The pre-publication history for this paper can be accessed here:



## Figures and Tables

**Table 1:** Relationship between TS and p53 expression, stratified by microsatellite instability status.

**MSI status**	**p53 status**	**TS status**		
			
		**Low**	**High**	**Total**
**All tumours**	**+**	86	158	244
	**-**	96	101	197
	
	**Total**	182	259	441*

**MSI tumours**	**+**	8	15	23
	**-**	21	21	42
	
	**Total**	29	36	65**

**MSS tumours**	**+**	78	143	221
	**-**	75	80	155
	
	**Total**	153	80	376***

*p = 0.005; **p = 0.30; ***p = 0.01; MSS, microsatellite stable; MSI, microsatellite instability; TS, thymidylate synthase

**Table 2:** Relationship between p53 expression and microsatellite instability status, stratified by TS expression.

**TS status**	**p53 status**	**MSI status**		
			
		**MSS**	**MSI**	**Total**
**All tumours**	**+**	221	23	244
	**-**	155	42	197
	
	**Total**	376	65	441*

**TS low level tumours**	**+**	78	8	86
	**-**	75	21	96
	
	**Total**	153	29	182**

**TS high level tumours**	**+**	143	15	158
	**-**	80	21	101
	
	**Total**	223	36	259***

*p = 0.001; **p = 0.03; ***p = 0.02; MSS, microsatellite stable; MSI, microsatellite instability; TS, thymidylate synthase

**Table 3:** Relationship between microsatellite instability status and TS expression, stratified by p53 expression.

**p53 status**	**MSI status**	**TS status**		
			
		**Low**	**High**	**Total**
**All tumours**	**MSS**	153	223	376
	**MSI**	29	36	65
	
	**Total**	182	259	441*

**p53 negative tumours**	**MSS**	75	80	155
	**MSI**	21	21	42
	
	**Total**	96	101	197**

**p53 positive tumours**	**MSS**	78	143	221
	**MSI**	8	15	23
	
	**Total**	86	158	244***

*p = 0.59; **p = 0.86; ***p = 1.0; MSS, microsatellite stable; MSI, microsatellite instability; TS, thymidylate synthase
